# Investigation on artificial blood or substitute blood replace the natural blood 

**Published:** 2014-04-20

**Authors:** Sh Keyhanian, M Ebrahimifard, M Zandi

**Affiliations:** 1 Associate Professor of Oncologist, Islamic Azad University of Tonekabon, Tonekabon, Iran; 2 Young Researchers and Elite Club, Tonekabon Branch, Islamic Azad University, Tonekabon, Iran; 3 Department of Biology, College of Sciences, Damghan branch, Islamic Azad University, Damghan, Iran

**Keywords:** Hematology, Artificial Blood, Perfluor, Hemoglobin, Blood Transfusion

## Abstract

Blood is a liquid tissue in which dissolved with abundant chemical factors and millions of different cells The reduction of unwanted side effects, especially diseases that emerge through blood such as HIV and hepatitis, has a significant role for modern medicine of transfusion and transplantation. The issues and costs of human blood collection and storage, direct this procedure towards the use of alternatives blood. Two important research fields of this area were oxygen carriers based on hemoglobin and perfluoro chemicals. While they do not have the same quality as the blood cell products, the oxygen carrier solutions have potential clinical and non-clinical applications.

The result showed that these products can reach to the body tissues easier than normal red blood cells, and can control the oxygen directly. The final aim of transfusion is to establish a transfusion system with no side effects, and the fact that oxygen carrier artificial blood has this property. The article attempts to step towards solving some problems of blood transfusion through describing the properties of artificial blood alternatives

## Introduction

There was a retry to adequate blood replacement so they could be kept in one category for any time or place without considering the blood type. Injection of any material other than autologous blood is the transfusions to replace the blood. So the history of blood transfusion can be considered as an alternative blood history (1,2,3). Substances like milk, casein derivatives, starch and saline were the first material to inject from one human to other human. Since World War II, the study of blood proposes was taken seriously to overcome large-scale situations like civilian events. Blood composition and functions of each replacement is clear except for portability oxygen (4,5).

This is a significant approach in two main replacements, means Hemoglobin and Perfolocarbon solutions (PFC).

Suggestions for allogeneic RBC have been an attempt to timely action against the adverse effects of blood transfusion. The risks involved in blood transfusion include infection, delayed wound healing associated with reactions transfusion, transfusion dependent chronic lung injury, and the potential recurrence of cancer of the immune system. Prevalence of HIV transmission by blood transfusion is about one in a million in5.2 million units of blood and, hepatitis C risk is one in 100,000 to one in 350,000 units. Non-infectious complications are associated with allogeneic transfusion. Some examples or for example, hemolytic transfusion reactions, transfusion-related lung diseases, diseases of organ transplants, and anaphylactic reactions, and purpura after they are transferred (6,7,8). Special techniques to reduce the need for blood transfusions have been developed during surgery. These techniques included the use of erythropoietin, drug treatment, surgical techniques and the minimum acceptable level of hemoglobin and blood substitutes (3,9).Currently, studies about blood transfusion are emphasized on Hemoglobin and Perfolocarbon solutions. (10)

**Fluorocarbon-based blood substitutes**

To modify human or animal hemoglobin is one of the ways to create a bloodsubstitute and thus help to solve the problem of lack of blood replacement the other way is to use materials better than hemoglobin. Thus, fluorocarbon chemicals were invented to replace the function of hemoglobin (11).

**Fluorocarbon compounds**

Liquid fluorocarbons are neutral chemical compounds in which hydrogen ions have been replaced by fluorine. They are sufficiently good solvents for oxygen and carbon dioxide to maintain the respiration of animal organisms (11,12,13).

Fluorocarbons are colorless, odorless,non-inflammable fluids of high density, stable in very high temperatures, entering poorly into chemical reactions, and poorly soluble. Their boiling point is low, as or because of their surface tension and viscosity. They differ in their molecular structure, boiling point, and oxygen solubility and the differences determine the variations in their biological action and in the time of their persistence

in the organism. For blood replacement and for systemic and organ perfusion fluorocarbons are used in the form of emulsions: FX80, FC75, FC43, FC17 (perfluorotetrahydrofuran, perfluorotributylamine) and lately also perfluorodecaline and perfluoromethyl decaline.

Fluorocarbon alone injected intravenously causes death of the animal since it does not mix with blood and produces pulmonary embolisnm, right ventricuilarfailure, and asphyxia. Sloviter et al showed that the pulmonary changes result from the production of platelet and not fluorocarbon emboli (11, 14). 

Fluorocarbonemulsion is usually well tolerated, although at the beginning of perfusion. They often cause a fail in arterial blood pressure. The emulsifiers usedincluding bovine albumin, lipids, and polyols for example, PlulronicF68, a mixture of polyoxypropylene and polyoxethylene.The emulsions are produced bythe use of mechanical energy or ultrasound, and selection of the right degree of dispersions of great importance for ensuring easy flow through the capillary vascular system and forobtaining the viscosity, gas diffusion capacity, and time of retention of the fluorocarbonin the circulation (14).

**Why fluorocarbon can be considered as an oxygen carrier?**

PFC is able to carry and release high volume oxygen to tissue, which is even more volume of physically dissolved gas relies on the solubility coefficient for that gas of the PFCs utilized, and is proportional to the gas’ partial pressure. This situation leads to several-fold increase of the oxygen concentration in PFCs emulsion by increasing oxygen concentration in the air inspired by the patient (15).

Because of the weak interaction between oxygen molecules and PFCs, the release ofO2 to tissues is greatly enhanced , and extraction rates and ratios are a lot higher withPFCs, which is able to reach 90% oxygen of its carrying, compared to only 25-30%for hemoglobin(15,16,17).

PFCs are non-reactive, and very stable for chemical properties.

**Hemoglobin-based oxygen carrier (HBOC)**

Hemoglobinis an obvious candidate for blood substitute that has a number of desirable features. The carrier has the ability to carry oxygen, and is countless of non-antigen complexes of RBC membrane (9,18,19). According to molecular stability improvements techniques, there are 4 groups of Hemoglobin cells. Surface modified hemoglobin - the molecule linked hemoglobin –polymerized hemoglobin- the hemoglobin liposomal capsule.HBOCs has half-life of 18 to 24 hours, which aresufficient for use in acute care. And they can be maintained for a period of 1-2 years in room of 4°C (21,24,25). The oxygen affinity of HBOCs is less than human’s major blood (26).

Traditionally it is recommended that for less than 600ml of blood, plasma expanders should be used. For 600-1200ml, red blood cells products are essential, and blood plasma derivatives or products such as low platelets or whole blood transfusion is critical. Blood substitutes should be used as a suggestion and / or complementary transfer of autologous or homologous or should be used in combination with Rito Protein (6, 27). 

**Human recombinant hemoglobin**

Hoffman and his colleagues described this human hemoglobin in E.coli in 1990. Luker and his colleagues in 1992 modulated the same hemoglobin as an artificial oxygen carrier, and generated rHb1.1. Α2β2 titrimetric hemoglobin is stabilized by the fusion of two α chain reducing renal toxicity associated with a titrimetric structure. The second generation of rHb2.0 hemoglobin has less NO than rHb1.1. But It’s production, despite favorable environmental effects as a side effect profile similar to DCLHb, were investigated by Boxer in 2003in a significant level (6,20,21,28,29,30)

**Raffimer hemoglobin**

Hemoglobin-based oxygen carrier hemoglobin Raffimer, Hemolicisoxidase from raffinoserelated to human blood were obtained by Mary Bridges. This hemoglobin covers only the remaining 40% tetrameric with only partial polymerization and has a half-life of one year. During the test phase 1 Hemolic

 From Kormicheal and his colleagues showed benefit,

but the inductor vasoconstriction, especially in people. In phase 2, 60 patients were studied, but there was a side effect of stress. In phase 3 transmission rate in the 299 patients with cardiac bypass surgery dropped 56% compared with 76% in the control group to indicate decreases. However, in a Phase 3 registration was suspended because it was increases in cardiac diseases. So Hemolic production ended in 2003 (31,32,33,34,35).

**Modified hemoglobin - activated polyethylene glycol - maleimide (MP4)**

MP4 was produced by the winslow&Sangret company. Hemoglobin molecules have been activated by surface interaction with polyethylene glycol maleimide. MP4 with high oxygen affinity of hemoglobin concentration was less 4/2g/dL of p50 was 2/5cp 60mmhg and high viscosity. (36,37,38).

**Bridge enzyme-linked hemoglobin**

Dismutase is an enzyme-linked hemoglobin product like this Catalase superoxide. They had advantages when used as oxygen carrier for the treatment of organ ischemic. Reperfusion in a mouse model confirmeda decreased production of oxygen free Redikar, but studies on animals or even humans do not (9,20,39,40).

**Stem cell hemoglobin**

Giaratana andcolleagues generated human RB adult stem cells developed hemoglobin. They contain the equivalent of hemoglobin and red blood cells in normal life. Stem cells are highper unit cost to produce the complexes (41).

**Future uses of perfluorocarbon emulsions**

Optimal use of perfluorocarbon emulsions in the futuremay consist of a combination of ANH preoperatively withapplication of an artificial oxygen carriersuch as a perfluorocarbonemulsion during the operation, a proceduretermed. Augmented ANH is a concept in which patients undergoANH to relatively low hemoglobin levels preoperatively. During the operation, when the hemoglobin concentrationdecreases further due to surgical blood loss and concomitant colloid or crystalloid replacement, perfluorocarbon emulsions in conjunction with 100% oxygen ventilationisadministered to enhance oxygen delivery andimprove tissue oxygenation. As a consequence, lowerlevels of hemoglobin concentration can be safely tolerated (12,25,42,43,44)

## Results

With the advance in bio-technology, there will be more blood substitutes available, which could permanently replace the natural blood. The substitute holds this kind ofpromise is artificial blood powder.

The ultimate goal of the transmission is the transmission system without any side effects and effective for the treatment. Although the current system of homologous blood has lack of issues, but it is much less expensive with fewer side effects, and is more acceptable. However, new technologies need to produce increasingly effective alternative for blood transfusion medicine. At present, the use of artificial blood due to short half-life and potential toxicity and costs, access to raw materials and the FDA is difficult. This caused temporary change to replace the red blood cells and the use of homologous blood transaction. Although the use of blood substitutes has higher safety incidence of viral infections like HIV, certainly in the near future, blood substitutes such as oxygen carrying will bring a new dimension in transfusion medicine.

**Table I:** Potential clinical applications of oxygen carrying solutions


1– Treatment • Blood substitutes : hemorrhagic shock; hemorrhage (war, surgery); anaemia. • Whole-body rinse out : acute drug intoxication; acute hepaticfailure. • Local ischemia: acute MI; evolving MI; cardiac failure; brain infarction; acute arterial thrombosis and embolism; PTCA ofcoronary artery. • General ischemia: gas embolism; CO intoxication; HAPO;HACO. • Aid for organ recovery : acute renal failure; acute hepatic failure;acute pancreatitis. • Infectious disease : anaerobic and aerobic diseaes;
**2**- Perfusional protection of organs during surgery – cardiopulmonarybypass, deep hypothermia, circulatory arrest, cardioplegia.
3- Preservation of donor organ.
**4**- Drug carrier - drug-conjugated haemoglobin and perfluorochemicals.
**5**- Contrast agent - (Perfluoro-octylbromide)
